# C-Fos expression is a molecular predictor of progression and survival in epithelial ovarian carcinoma

**DOI:** 10.1038/sj.bjc.6604650

**Published:** 2008-10-14

**Authors:** S Mahner, C Baasch, J Schwarz, S Hein, L Wölber, F Jänicke, K Milde-Langosch

**Affiliations:** 1 Department of Gynecology and Gynecologic Oncology, University Medical Center Hamburg-Eppendorf, Martinistrasse 52, 20246 Hamburg, Germany

**Keywords:** ovarian cancer, prognostic factors, c-Fos, AP-1, survival, progression

## Abstract

Members of the Fos protein family dimerise with Jun proteins to form the AP-1 transcription factor complex. They have a central function in proliferation and differentiation of normal tissue as well as in oncogenic transformation and tumour progression. We analysed the expression of c-Fos, FosB, Fra-1 and Fra-2 to investigate the function of Fos transcription factors in ovarian cancer. A total of 101 patients were included in the study. Expression of Fos proteins was determined by western blot analysis, quantified by densitometry and verified by immunohistochemistry. Reduced c-Fos expression was independently associated with unfavourable progression-free survival (20.6, 31.6 and 51.2 months for patients with low, moderate and high c-Fos expression; *P*=0.003) as well as overall survival (23.8, 46.0 and 55.5 months for low, moderate and high c-Fos levels; *P*=0.003). No correlations were observed for FosB, Fra-1 and Fra-2. We conclude that loss of c-Fos expression is associated with tumour progression in ovarian carcinoma and that c-Fos may be a prognostic factor. These results are in contrast to the classic concept of c-Fos as an oncogene, but are supported by the recently discovered tumour-suppressing and proapoptotic function of c-Fos in various cancer types.

Ovarian cancer accounts for the highest tumour-related mortality among women with gynaecologic malignancies. The American Cancer Society estimates about 23 000 new cases of ovarian cancer each year in the United States and that 70% of the affected women will die from their disease (American-Cancer-Society, 2007). Although aggressive surgical cytoreduction and platinum-based combination chemotherapy have improved outcome for many patients, long-term survival could not generally be improved. Identification of additional prognostic factors could help to stratify patients into different biological subgroups. In ovarian cancer, this is especially important for the group of patients with early relapse (within 6 months after first-line treatment) that usually die within 6–12 months (du Bois *et al*, 2003). These patients (approximately 25%) do not benefit from current treatment modalities while suffering from the sometimes severe side effects of therapy. Subsequent research could then focus on the establishment of more targeted and individual treatment strategies in this subgroup, as previously shown for Her2/neu expression and trastuzumab treatment in breast cancer (Pegram *et al*, 2000).

Widely accepted prognostic factors in patients with epithelial ovarian cancer are International Federation of Gynecology and Obstetrics (FIGO) stage and residual tumour volume after primary surgical cytoreduction (Bristow *et al*, 2002; Tingulstad *et al*, 2003). Several other clinical and biological factors such as age, performance status, tumour histology and grade have been assessed for prognostic significance over the past decades, but none of them yielded conclusive and reproducible results (Omura *et al*, 1991; Hornung *et al*, 2004; Winter *et al*, 2007).

Recent efforts to develop accurate predictors of clinical outcome have mainly focused on assessment of global gene expression by DNA microarrays. This technology provided information on differential gene expression in a number of tumours including ovarian cancer (Hartmann *et al*, 2005) and has identified gene profiles associated with early relapse and decreased survival (Spentzos *et al*, 2004). Members of the Fos family (c-Fos, FosB, Fra-1 (Fos-related antigen 1) and Fra-2) are often represented in these profiles (Meinhold-Heerlein *et al*, 2005). They dimerise with the gene products of c-Jun, JunB or JunD to form the transcription factor Activating Protein 1 (AP-1). AP-1 binds to the promoter region of specific target genes, converting extracellular signals into changes of gene expression (Milde-Langosch, 2005). As a member of AP-1, c-Fos has been implicated mainly in signal transduction, cell differentiation and proliferation (Shaulian and Karin, 2001). Many studies focused on its oncogenic functions and found that c-Fos regulated genes important for tumorigenesis, causing the downregulation of tumour-suppressor genes (Bakin and Curran, 1999) and leading to invasive growth of cancer cells (Hu *et al*, 1994). Furthermore, c-Fos can induce a loss of cell polarity and epithelial-mesenchymal transition, leading to invasive and metastatic growth in mammary epithelial cells (Fialka *et al*, 1996).

In addition to these experimental results, several reports investigated the function of c-Fos expression in human tumour tissue. In osteosarcoma and endometrial carcinoma, c-Fos overexpression was associated with high-grade lesions and adverse outcome (Gamberi *et al*, 1998; Bamberger *et al*, 2001). In a comparative analysis between precancerous lesion of the cervix uteri and invasive cervical cancer, c-Fos expression was significantly lower in precancerous lesions (Prusty and Das, 2005). C-Fos has also been identified as independent predictor of decreased survival in breast cancer (Bland *et al*, 1995).

However, some more recent studies have raised the idea that c-Fos may also have tumour-suppressor activity and might have a function in apoptosis (Teng, 2000). Overexpression of c-Fos was found to inhibit cell cycle progression, stimulated murine hepatocyte cell death and strongly suppressed tumour formation *in vivo* (Mikula *et al*, 2003). A functional involvement of c-Fos in apoptosis has been shown by its regulatory involvement during remodelling and stress response in various tissues in mouse development (Jochum *et al*, 2001).

Besides c-Fos, FosB, Fra-1 and Fra-2 have also been shown to have a function in progression of various tumour types: FosB is downregulated in poorly differentiated mammary carcinomas (Bamberger *et al*, 1999), whereas Fra-1 and, partly, Fra-2 overexpression leads to enhanced tumour cell motility and invasion in breast cancer, colorectal cancer and mesothelioma (Milde-Langosch, 2005).

This study investigated the potential function of Fos transcription factors in ovarian cancer and analysed the expression and prognostic significance of c-Fos, FosB, Fra-1 and Fra-2 in patients with invasive epithelial ovarian carcinoma.

## Materials and methods

### Patients

Patients with epithelial ovarian carcinoma who presented for primary surgery at the University Medical Center Hamburg-Eppendorf between 1997 and 2006 were included in this study. Thus, a total of 101 patients were retrospectively analysed. Detailed patient characteristics are listed in Table 1. Clinicopathologic factors were evaluated by reviewing medical charts and pathologic records. Tissue slides were reviewed for histological classification and clinical outcome was followed from the date of surgery to the date of death or until the end of 2007. Two cases were lost to follow-up right after surgery and were excluded from the survival analysis. All patients gave written informed consent to access their tissue and review their medical records according to our investigational review board and ethics committee guidelines.

### Tissue samples

Fresh-frozen samples were obtained intraoperatively and immediately stored at −80°C. The histological characteristics of each sample were assessed on cryo-cut and haematoxylin-eosin-stained sections and the tissue was trimmed if necessary to obtain at least 70% tumour cells in the sample used for protein extraction.

### Protein extraction

Samples of approximately 100 mg were cut from the tissue and pulverised using a micro-dismembrator (Braun-Melsungen, Melsungen, Germany) for 2 × 45 s at 200 r.p.m. Proteins were lysed in ice-cold sample buffer (50 mM Tris pH 6.8, 1% sodium dodecyl sulphate (SDS), 10% sucrose and 10 *μ*l ml^−1^ protease inhibitor cocktail (Sigma, Taufkirchen, Germany)), and protein concentration was determined following the standard protocols and using bovine serum albumin protein standards as described previously (Bamberger *et al*, 1999; Milde-Langosch *et al*, 2005).

### Western blot analysis

Equal amounts of protein (20 *μ*g) of each sample were loaded per well, and equal loading was verified by immunoblotting with actin antibodies (Santa Cruz, Heidelberg, Germany). As control samples, proteins from the ovarian cancer cell lines Ovcar5 and Ovcar8 and the breast cancer cell line MCF7 were loaded on each gel. After electrophoresis, blotting to polyvinylidene difluoride membranes and overnight incubation at 4°C in blocking solution, membranes were incubated for 1 h at room temperature with the following primary antibodies (all from Santa Cruz): c-Fos polyclonal antibody no. 4 (1 : 1000), FosB polyclonal antibody no.102 (1 : 200), Fra-1 polyclonal antibody no. R-20 (1 : 400) and Fra-2 polyclonal antibody no. Q-20 (1 : 800). As secondary antibody, peroxidase-conjugated anti-rabbit-IgG (1 : 4000) was used, which was visualised by chemiluminescence reagent (Super Signal West Pico kit, Pierce, Rockfort, IL, USA) using Hyperfilm ECL films (Amersham, Braunschweig, Germany). Band intensities were quantified by densitometry (GS-700 Imaging Densitometer, BioRad, Munich, Germany). Intensities of the specific protein bands were calculated as percent intensity of the control sample and corrected for equal actin loading as previously described (Bamberger *et al*, 1999).

### Immunohistochemistry

Serial sections of 4–6 *μ*m were deparaffinised in xylene, rehydrated and microwaved for 20 min in 20 mM Tris, 10 mM citrate, 13 mM EDTA, pH 7.8. After cooling down for 20 min, the slides were washed in TBS (50 mM Tris, 150 mM NaCl, pH 7.4), blocked for 30 min at room temperature with normal serum (rabbit IgG, ABC Kit, Vector Laboratories, Burlingale, CA, USA), diluted 1 : 20 in TBS and then incubated overnight at 4°C with the same c-Fos antibody used for western blots, diluted 1 : 100. After washing, slides were reacted with biotin-labelled anti-mouse or anti-rabbit immunoglobulin (IgG), incubated with preformed ABC-complex (Vectastain, Vector Laboratories) and detected with DAB-substrate kit (Vectastain, Vector Laboratories). The slides were counterstained with hematoxylin. For negative controls the primary antibody was omitted.

### Statistical analysis

The *χ*^2^ test and Fisher's exact test (two-sided) were used to examine the correlation between the expression of Fos proteins and clinicopathologic factors (age, FIGO-stage, histology, grade, CA-125). For statistical analysis, the cases were divided into three equal groups representing low, moderate and high expression of the analysed transcription factor. These groups were compared with the clinicopathological factors tumour stage (FIGO I/II *vs* III *vs* IV), residual tumour after surgery (<1 *vs* ⩾1 cm), grading (G1/G2 *vs* G3), age (<65 *vs* ⩾65 years), histological subtype (serous *vs* endometrioid/mucinous *vs* others) and CA125 serum level (below *vs* above median). Survival curves were plotted using the Kaplan–Meier method and differences between survival curves were tested using the log-rank test. For multivariate analysis, Cox regression analysis was performed. Probability values less than 0.05 were regarded as statistically significant. All statistical analyses were conducted using SPSS software Version 15 (SPSS Inc., Chicago, IL, USA).

## Results

### Patients

A total of 101 patients were included in this study; detailed characteristics are listed in Table 1. All patients underwent radical surgery including hysterectomy, bilateral salpingo-oophorectomy, appendectomy, infragastric omentectomy and systematic pelvic and paraaortic lymphadenectomy as well as resection of all visible tumour. In the majority of patients, optimal debulking could be achieved (67 patients with microscopic residual tumour and 17 patients with residual tumour <1 cm). Ninety-six patients received platinum-based first-line chemotherapy, predominantly in combination with a taxane; six patients were treated with 2–3 preoperative (neoadjuvant) cycles of chemotherapy as part of a phase II trial. Median follow up time was 20 months.

In the study cohort, progression-free survival ranged between 0.4 and 98 months with a median of 15.2 months; median overall survival was 20 months and ranged from 0.4–98 months.

### Expression of c-Fos, FosB, Fra-1 and Fra-2 in ovarian carcinomas

**Figure 1:**
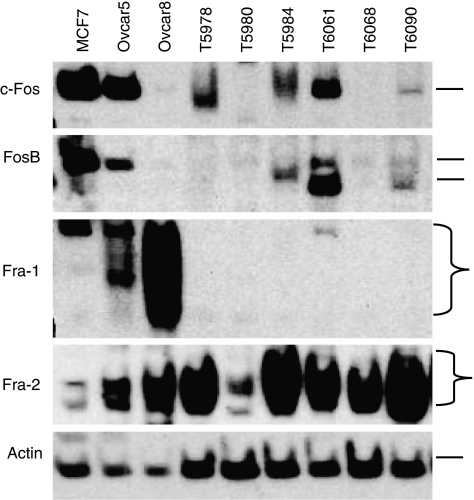
Representative results of c-Fos, FosB, Fra-1 and Fra-2 expression in ovarian carcinomas. As control, protein extracts from the ovarian cancer cell lines Ovcar5 and Ovcar8 as well as the mammary carcinoma cell line MCF7 were included in each gel. Tumour samples were coded as Txxxx and equal amounts of protein (20 *μ*g) were loaded on the gel.

A representative western blot analysis of c-Fos, FosB, Fra-1 and Fra-2 expression is shown in Figure 1. As control, proteins extracted from the ovarian cancer cell lines Ovcar5 and Ovcar8 as well as the mammary carcinoma cell line MCF7 were included in each gel.

C-Fos expression varied extensively in different samples with a strong signal at around 55 kDa in MCF7 and Ovcar5 cells and some carcinomas, whereas the signal was only weak or undetectable in Ovcar8 cells and other tumours. Compared to the expression in Ovcar5 cells, which was defined as 100%, c-Fos expression ranged between 0.8 and 283% (mean 38.5%, median 21.3%) in the tumour samples.

**Figure 2:**
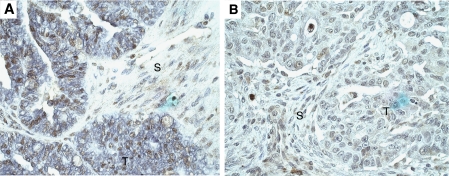
C-Fos immunohistochemistry. (**A**) Moderately differentiated serous carcinoma with nuclear immunoreactivity in tumour cells (T) and weak immunostaining in nuclei of some stromal fibroblasts (S). 400 × . (**B**) Poorly differentiated serous carcinoma with only weak c-Fos immunostaining in tumour cells (T) and some fibroblasts (S). 400 × .

As the protein extracts used in this study contained not only carcinoma cells, but also varying portions (<30%) of stromal fibroblasts, we performed immunohistochemistry with paraffin sections of 14 tumours to find out which cells are expressing the c-Fos protein. In most cases, nuclear c-Fos immunostaining was found in 2–50% of tumour cells (Figure 2), but cytoplasmic staining was also seen in some cases. In addition, weak-to-moderate nuclear c-Fos reactivity was observed in 10–50% of stromal fibroblasts.

FosB was detected as 1 or 2 bands at 48–55 kDa, with high expression in Ovcar5 and MCF7 cells, low protein expression in Ovcar8 cells and strong variations in the tumour samples. Results of densitometry ranged between 2 and 2307% when FosB expression in Ovcar5 was set as 100% (mean 236%, median 112%). The smaller splice product FosB2 was detected on the same membranes as a weak signal in most tumours (not shown).

Fra-2 was strongly expressed in Ovcar8 cells and most ovarian tumours. Owing to posttranslational phosphorylation, it was detected as 2–4 bands at 38–47 kDa. Compared to Ovcar8, the mean expression level after densitometry was 154% (range 5–480%, median 130%).

Fra-1 expression was extremely strong in Ovcar8 cells, but relatively weak in the analysed tissue samples. Owing to background staining and only weak Fra-1-specific bands, densitometric evaluation of band intensity could not be performed. Instead, Fra-1 expression was scored semiquantitatively as negative (*n*=8), low (*n*=47), moderate (*n*=27) or strong (*n*=19).

### Correlation of Fos protein expression with clinicopathological parameters

Correlations between Fos protein expression and clinicopathological factors are listed in Table 2. A statistically significant correlation could be observed between expression of c-Fos and FosB. Loss of c-Fos expression was significantly more frequent in high-grade carcinomas. Expression of FosB correlated with Fra-1 expression, but no statistically significant correlations could be observed for FosB, Fra-1, Fra-2 and the other clinicopathologic factors evaluated.

For the FosB splice variant FosB2 and single Fra-1 and Fra-2 bands representing the differentially phosphorylated proteins, a separate densitometric evaluation and statistical analysis was performed as described for the other Fos proteins. However, no significant correlations with histological or clinicopathological factors were found (not shown).

### Correlation of c-Fos expression with progression-free survival and overall survival

**Figure 3:**
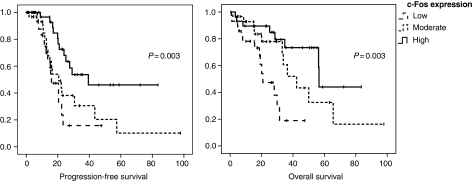
C-Fos expression is correlated with progression-free and overall survival (*P*=0.003). Kaplan–Meier curves were generated from 99 patients whose outcome was followed over a median period of 20 months. Patients were stratified based on low, moderate and high c-Fos expression. *X* axis: survival probability; *Y* axis: survival (months). Censored cases are indicated by vertical bars.

Reduced c-Fos expression was associated with significantly shorter progression-free survival (20.6, 31.6 and 51.2 months for patients with low, moderate and high c-Fos expression; *P*=0.003). The same could be observed for overall survival with 23.8, 46.0 and 55.5 months for low, moderate and high c-Fos levels (*P*=0.003, Figure 3). These results did not change, when patients with neoadjuvant chemotherapy (*n*=6) were excluded from the analysis. For FosB, Fra-1 and Fra-2, no associations with survival were found (not shown). In addition to the prognostic function of c-Fos, univariate analysis revealed a statistically significant impact on survival for CA-125 level before surgery, histological subtype, FIGO-stage and residual tumour after surgery (Table 3).

Subsequently, a multivariate Cox regression analysis including c-Fos, residual tumour, FIGO-stage, histological subtype and preoperative CA-125 serum levels was performed. Reduced c-Fos expression, residual tumour ⩾1 cm and advanced FIGO-stage were associated with significantly shorter progression-free survival. Furthermore, c-Fos expression and residual tumour were independently associated with poor overall survival (Table 4).

## Discussion

To investigate the function and prognostic significance of Fos-proteins in epithelial ovarian carcinoma, we analysed the expression of c-Fos, FosB, Fra-1 and Fra-2 in 101 patients with invasive epithelial carcinoma of the ovary. We could demonstrate for the first time that loss of c-Fos expression correlates with disease progression and c-Fos might be an independent prognostic factor in ovarian carcinoma.

The results of our study are in contrast to the mainstream opinion concerning the oncogenic function of c-Fos, but an increasing number of recent experimental and clinical reports support our findings (Teng, 2000). Growing evidence from *in vitro* and *in vivo* studies suggests that c-Fos might actually be able to do both, promote and suppress tumorigenesis. This double action could be enabled by differential protein composition of tumour cells and their environment, for example, dimerisation partners, co-activators and promoter architecture.

Decreased c-Fos expression was observed in metastatic mammary carcinoma cell lines compared to non-metastatic cells (Kustikova *et al*, 1998). In tissue samples of human non-small cell lung cancer and thyroid carcinoma, c-Fos expression was significantly lower compared to normal tissue (Levin *et al*, 1995; Liu *et al*, 1999). Recently, an immunohistochemical study including more than 600 patients with gastric carcinoma could demonstrate that loss of c-Fos expression was associated with adverse outcome (Jin *et al*, 2007).

One possible explanation for the tumour-suppressor activity of c-Fos could be a proapoptotic function, which might confer increased chemoresistance to tumours with low c-Fos protein levels. Induction of c-Fos results in apoptosis in murine hepatocytes that conditionally express c-Fos (Mikula *et al*, 2003). *Fos*^−/−^*tp53*^−/−^ double-knockout mice develop highly invasive and proliferative rhabdomyosarcoma, a tumour rarely observed in *tp53*^−/−^ knockout-mice (Fleischmann *et al*, 2003). Of note, re-expression of c-Fos in an established tumour cell line from these mice increased apoptosis. The exact mechanisms by which c-Fos contributes to apoptosis are poorly understood. Elevated c-Fos expression is associated with concomitant activation of the AP-1 transcription factor complex (Schadendorf *et al*, 1996; Schaerli and Jaggi, 1998; Huang *et al*, 2003). AP-1 activity has been mainly associated with cell proliferation and tumour progression, but there is increasing evidence that AP-1 might also have an important function in cell death (Shaulian and Karin, 2001). The ability of AP-1 to participate in several different cellular processes requires activation of different target genes under different conditions. It is possible that changes in the composition of AP-1 are essential in cellular response to different stimuli.

Observations in human hepatocellular carcinoma cells indicate that c-Fos is a mediator of c-myc-induced cell death and might induce apoptosis through the p38 MAP kinase pathway (Kalra and Kumar, 2004). Fas ligand (FASLG or FasL) and the tumour necrosis factor-related apoptosis-inducing ligand (TNFSF10 or TRAIL) might reflect an additional apoptotic mechanism induced by c-Fos, as observed in a human T-cell leukaemia cell line (Siegmund *et al*, 2001). Another possible mechanism of c-Fos involvement in tumour suppression could be the direct regulation of BRCA1, a well established factor in familial breast and ovarian cancer (Graves *et al*, 2007).

In ovarian cancer, indirect evidence of a possible antioncogenic function of c-Fos has recently been described. Appierto *et al*., (2004)could observe an involvement of c-Fos in fenretinide-induced apoptosis in human ovarian carcinoma cells. In their study, apoptosis was accompanied by elevation of c-Fos expression at mRNA and protein level. This observation supports our results, which demonstrate an association of reduced c-Fos expression with adverse outcome (Figure 3, Tables 3 and 4).

The correlation between low-grade tumours and high c-Fos expression that we observed could also be demonstrated in a molecular comparison between serous ovarian carcinomas of varying grade and borderline (low malignant potential, LMP) tumours (Meinhold-Heerlein *et al*, 2005). In that series, c-Fos mRNA levels were significantly higher in LMP and low-grade tumours compared to intermediate and high-grade carcinomas. Analogue findings were observed in a comparative analysis of gene expression profiles in normal and neoplastic ovarian tissue samples (Welsh *et al*, 2001): c-Fos was highly expressed in normal tissues and weakly expressed in tumours. Although normal tissues and LMP tumours were not included in our study, we observed the same results for invasive carcinomas of varying grade (Table 2).

The improved outcome we noticed with increased expression of c-Fos could also imply that ovarian carcinomas are more susceptible to treatment, when c-Fos is activated. This hypothesis is supported by the observation that patients with advanced colorectal carcinoma had higher response rates to cytostatic treatment and improved survival, when intratumoral c-Fos expression was high (Singh *et al*, 1998). However, the results of our study cannot determine whether the positive prognostic effect of c-Fos is the result of intrinsic differences in malignant potential of the tumours or of differences in chemo-resistance.

Findings of previous experimental studies suggested that Fra-1 expression might also have a function in ovarian cancer (Hapke *et al*, 2003; Tchernitsa *et al*, 2004). However, our results could not show an impact of Fra-1, Fra-2 and FosB on ovarian cancer progression.

Limitations of our study are its retrospective monocentric nature and the fact that tumour tissue was not strictly collected in consecutive patients, leading to a possible selection bias. However, the high number of patients with optimal surgical cytoreduction and thorough surgical staging followed by platinum-based combination chemotherapy might be a strength of this study: In the presence of large residual tumours after primary surgery, progression mainly occurs due to selection of resistant tumour clones during first-line chemotherapy. Biological factors leading to true primary chemo-resistance and progression are more likely to have a function if only microscopic residual tumour is present.

In summary, this report documents that loss of c-Fos expression is significantly and independently associated with reduced progression-free and overall survival in a cohort of optimally treated patients with epithelial ovarian cancer. These results suggest for the first time that c-Fos might have a function in tumour suppression in ovarian cancer. It may be clinically useful for stratification of platinum-resistant patients but additional studies are needed to confirm our findings and assess the underlying molecular mechanisms.

## Figures and Tables

**Table 1:** Patient characteristics

**No. of patients**	**101**
*Age (years)*	
Mean	59.2
Median	61
Range	21–87
	
*FIGO stage*	
I	10
II	8
III	60
IV	23
	
*Grading*	
1	6
2	29
3	58
Not determined/unknown	8
	
*Lymph node metastasis*	
N0	31
N1	55
NX	15
	
*Residual tumour after surgery*	
Microscopic	67
<0.5 cm	7
0.5–1 cm	10
1–2 cm	2
>2 cm	6
Not determined/unknown	9
	
*Histologic subtype*	
Serous	74
Mucinous	6
Endometrioid	5
Clear cell	1
Undifferentiated	7
Mixed differentiation	8
	
*Platinum-based first-line chemotherapy*	
Adjuvant	90
Neoadjuvant	6
None/unknown	5
	
*CA 125 before surgery (kU l*^−*1*^)	
Mean	2178
Median	436
Range	21–47 000
	
*Survival (months), n*=*99*	
Progression-free survival	
Mean	19.7
Median	15.2
Range	0.4–98
	
Overall survival	
Mean	24.4
Median	20
Range	0.4–98

FIGO=International Federation of Gynecology and Obstetrics; kU/l=kilo Units per liter.

**Table 2:** Correlations of Fos proteins with clinical/pathological variables (*P*-values) and with each other

	**c-Fos**	**FosB**	**Fra-1**	**Fra-2**
Age	0.431	0.715	0.167	0.139
Grading	0.038^a^	0.149	0.384	0.469
FIGO stage	0.586	0.162	0.709	0.633
Histologic Subtype	0.393	0.206	0.674	0.372
CA-125	0.911	0.306	0.881	0.062
				
FosB	0.001	—	—	—
Fra-1	0.058	0.003	—	—
Fra-2	0.403	0.095	0.593	—

a Inverse correlation.

**Table 3:** Univariate Kaplan–Meier analysis of clinicopathological factors in relation to progression-free survival (A) and overall survival (B)

	**Progression-free survival**	**Overall survival**
**Factor**	***P*-value**	***P*-value**
Age	0.415	0.92
CA-125	0.008	0.082
Histology	0.016	0.039
FIGO stage	0.001	0.048
Residual tumour	<0.001	<0.001
Grading	0.475	0.896

FIGO=International Federation of Gynecology and Obstetrics.

**Table 4:** Multivariate Cox regression analysis including c-Fos expression, residual tumour, FIGO stage, histologic subtype, and preoperative CA-125 serum levels to determine the impact on progression-free survival (A) and overall survival (B)

	**Progression-free survival**		
**A**	**HR**	**95% CI**	***P*-value**
c-Fos	0.554	0.353–0.870	0.010
Residual tumour	4.824	1.226–18.990	0.024
FIGO stage	1.917	1.013–3.628	0.046
Histology	1.032	0.548–1.942	0.922
CA-125	1.936	0.888–4.222	0.097
			
	**Overall survival**		
**B**	**HR**	**95% CI**	***P*-value**
c-Fos	0.546	0.310–0.961	0.036
Residual tumour	6.022	1.697–21.369	0.005
FIGO stage	1.390	0.589–3.277	0.452
Histology	1.048	0.493–2.228	0.903
CA-125	1.589	0.637–3.967	0.321

CI=confidence interval; FIGO=International Federation of Gynecology and Obstetrics; HR=hazard ratio.
